# TTR sequencing should be considered ahead of hypertrophic cardiomyopathy in Afro-Americans

**DOI:** 10.1186/1750-1172-10-S1-P35

**Published:** 2015-11-02

**Authors:** Jean Herlé Raphalen, Mathieu Kerneis, Xavier Waintraub, Riadh Cheikh-Khelifa, PIerre Fouret, Philippe Cluzel, Zahir Amoura, Fleur Cohen Aubart

**Affiliations:** 1 Pitié-Salpêtrière Hospital, Internal Medicine Department, e3m Institute, French National Reference Center for Rare Systemic diseases, 75013, Paris, France; 2 Pitié-Salpêtrière Hospital, Cardiology Department, 75013, Paris, France; 3 Pitié-Salpêtrière Hospital, Anatomo-pathology Department, 75 013, Paris, France; 4 Pitié-Salpêtrière Hospital, Cardiovascular and Interventional Imaging Department, 75 013, Paris, France

## Background

Amyloid cardiomyopathy is a polymorphic condition with heterogeneous prognosis. Whereas AL amyloid cardiomyopathy is the most frequent type of amyloid cardiopathy, transthyretin (TTR) amyloidosis is often under diagnosed. TTR gene sequencing may be easily performed although usually used after a histological proof of amyloidosis is obtained. We conducted a prospective study to evaluate the interest of TTR gene sequencing in hypertrophic cardiomyopathy with suspicion of amyloidosis before obtaining a histological proof of amyloidosis.

## Methods

All patients referred for hypertrophic cardiomyopathy between January 2014 and April 2015 with suspicion of amyloidosis on echocardiography or cardiac MRI were screened with light chain dosage, protein electrophoresis and C-reactive protein dosage. When these exams were normal, they were included in the study and underwent TTR gene sequencing.

## Results

Eight patients were included in the study, 7 men and 1 woman, median age at the time of inclusion 74 years. All patients had clinical signs of congestive heart failure with elevated NT-proBNP levels. No familial history was noted. Among them, 4 patients, all from carribean origin, were diagnosed as having hereditary TTR cardiac amyloidosis by sequencing of the TTR gene. These 4 patients had hypertrophic cardiomyopathy with altered diastolic and systolic function, diagnosed respectively 7, 3, 2 years and six months before. Previous exams had eliminated ischemic and valvular cardiomyopathy. Echocardiographic study revealed increase of left ventricular wall thickness predominant on septum wall with shiny appearance. LV ejection fraction was between 35 and 50 %. Two out the 4 patients underwent cardiac MRI which showed diffuse delayed gadolinium enhancement suggestive of amyloidosis. As all the patients included in the study, these 4 patients did not have monoclonal gammapathy and light chain dosage was normal. Transthyretin sequencing revealed the presence of a missense mutation Val122Ile (c.424 G>A) in these 4 patients. One of the patients underwent a cardiac transplantation and pathological examination confirmed amyloid cardiopathy. Only 1 other patient had a confirmation of amyloid deposit after an extensive work-up (patient #8, see below).

Among the 8 patients, 8 had minor salivary gland biopsy which revealed presence of amyloidosis deposits in only 1 (patient #8, with a Val122Ile TTR mutation), 3 had abdominal fat aspiration (normal in all), 2 underwent rectal mucosal biopsy (normal in all). Among the 4 patients who did not have a TTR mutation, 3 underwent a endomyocardial biopsy to confirm the amyloid nature of the cardiopathy which was positive in all 3. Final diagnosis was AA amyloidosis for one, senile TTR for one and non-typed amyloidosis for the last one.

## Conclusion

TTR sequencing is a specific, non-invasive way to diagnose TTR amyloid cardiomyopathy and should be considered after exclusion of alternative causes of hypertrophic cardiopathy.

